# Supplementary material for: HaplotagLR: An efficient and configurable utility for haplotagging long reads
Source: PLoS One. 2024 Mar 13;19(3):e0298688. doi: 10.1371/journal.pone.0298688 (PMC10936807; doi:10.1371/journal.pone.0298688)
Supplement: Attachment — Submitted filename: LRphase_PLOS-One_rebuttal-letter.rev1.docx [file pone.0298688.s002.docx]

Black text: Reviewers' comments
Red text: Authors’ responses

Reviewer's Responses to Questions

**Comments to the Author**

1. Is the manuscript technically sound, and do the data support the conclusions?

The manuscript must describe a technically sound piece of scientific research with data that supports the conclusions. Experiments must have been conducted rigorously, with appropriate controls, replication, and sample sizes. The conclusions must be drawn appropriately based on the data presented.

Reviewer #1: Partly

We have attempted to address the issues raised with appropriate changes and additional data added to the main text and supplement, as indicated alongside the relevant comments in the detailed remarks.

Reviewer #2: Yes

2. Has the statistical analysis been performed appropriately and rigorously?

Reviewer #1: Yes

Reviewer #2: No

We were, unfortunately, unable to respond to Reviewer 2’s concerns about statistical rigor as no specific concerns were brought up in the detailed comments. Accordingly, we have reviewed all statistics reported in the manuscript and could find no obvious errors or omissions in the data or in reporting of test types, parameters, significance thresholds, etc.

3. Have the authors made all data underlying the findings in their manuscript fully available?

The [PLOS Data policy](https://nam02.safelinks.protection.outlook.com/?url=http%3A%2F%2Fwww.plosone.org%2Fstatic%2Fpolicies.action%23sharing&data=05%7C01%7Cadadiehl%40med.umich.edu%7C0aa22c2ef5174fe93a9508dbc356ea66%7C1f41d613d3a14ead918d2a25b10de330%7C0%7C0%7C638318550847931109%7CUnknown%7CTWFpbGZsb3d8eyJWIjoiMC4wLjAwMDAiLCJQIjoiV2luMzIiLCJBTiI6Ik1haWwiLCJXVCI6Mn0%3D%7C3000%7C%7C%7C&sdata=fYDSvHz82q2nVi4vHr8j9XA2Kk1Duqulepy7X9ZhY%2Fw%3D&reserved=0) requires authors to make all data underlying the findings described in their manuscript fully available without restriction, with rare exception (please refer to the Data Availability Statement in the manuscript PDF file). The data should be provided as part of the manuscript or its supporting information, or deposited to a public repository. For example, in addition to summary statistics, the data points behind means, medians and variance measures should be available. If there are restrictions on publicly sharing data—e.g. participant privacy or use of data from a third party—those must be specified.

Reviewer #1: No

It appears the main concern is a lack of easy access to helper scripts and parameter values used in fixing GIAB VCF files and data simulation. In response, we have added the corresponding script, and a simple shell script to perform these operations with the same parameters used in the manuscript analysis. New link is https://zenodo.org/record/7819303#.ZDW6sexudzU.

Reviewer #2: Yes

4. Is the manuscript presented in an intelligible fashion and written in standard English?

PLOS ONE does not copyedit accepted manuscripts, so the language in submitted articles must be clear, correct, and unambiguous. Any typographical or grammatical errors should be corrected at revision, so please note any specific errors here.

Reviewer #1: Yes

Reviewer #2: Yes

5. Review Comments to the Author

Please use the space provided to explain your answers to the questions above. You may also include additional comments for the author, including concerns about dual publication, research ethics, or publication ethics. (Please upload your review as an attachment if it exceeds 20,000 characters)

Reviewer #1: The authors present a conceptionally simple statistical approach and a corresponding tool LRPhase to assign long reads to haplotypes defined by a given set of phased variants. This process is called haplotagging. LRPhase allows to specify an FDR and thus to adjust specificity and sensitivity according to application.

The manuscript is well written and the aim of haplotagging and potential application areas are well described. LRphase is applied on simulated data from Genome in a Bottle (GIAB) and on real data (ONT sequencing data). Comparison was performed with the haplotag mode of whatshap using precision-recall (PR) curves and PR-AUC on one simulated data set and percentages of haplotagged reads for one real data set.

The authors made an effort to provide data in reproducible and convenient ways using SRA, Github, Zenodo and Bioconda.

*All data are available except helper scripts for file fixing and data simulation.* Although the corresponding analysis steps are explained in detail in the manuscript, I strongly encourage providing these scripts as well.

Scripts for these operations are now included in the Zenodo archive at <https://zenodo.org/record/8436101>. The Zenodo link within the text has been updated accordingly. There is no separate script for producing the simulated data – issuing the happlotagLR command provided in-text will recreate the read simulation process as reported in the text. Since no random seed was specified in simulations, we cannot guarantee this will produce identical reads, thus we also provide the simulated bams used in this study as part of the archive.

I think the described model and tool is useful, since haplotagging, i.e. read phasing according to a given set of phased variants, will gain importance as long-read sequencing is used not only for variant phasing and assembly, but increasingly also for the generation of downstream information such as targeted deep sequencing or RNA sequencing. Particularly, improving models to consider base calling error rates, as done here, as well as reporting characteristic numbers reflecting the specific result and quality of individual phased reads can be helpful in some application settings. However, there are some possible major shortcomings that need to be addressed. Further, there are missing results and information that will need to be added to put the work in relation to existing work and to make sure that the current version of the tool is indeed capable of addressing the issues discussed in the manuscript. I will start with a conceptional issue that I think needs to be addressed in the manuscript followed by further major concerns and corresponding suggestions for improvements:

• As clearly explained in the manuscript, haplotagging can be considered the reverse operation of read-based phasing. Thus, both operations are tightly linked, which is also the reason, why there are to date, to my knowledge, no tools dedicated solely to haplotagging.

This comment and others raising multiple issues will be broken point by point…

- Typically, a phased VCF is the result from long read-based phasing (in the most accurate setting).

This is correct, particularly in a precision medicine setting or when working with cell lines that are not already well-characterized and annotated with high-confidence variants. However, performing read-based phasing start to finish may not be necessary in order to perform haplotagging-based analyses in well-studied cell lines, such as the NA12878 cells employed in the present study. We have added relevant information to the introduction to clarify (lines 63-67, e.g.)

- Why would additional long-read data to be haplotagged not be used to improve the phased variant calls or to generate phased variant calls? Newly called and phased variants could simply be compared to the phased variants obtained elsewhere, or sequencing data combined to improve variant phasing. This is the common setting addressed by whatshap.

A standalone haplotagging utility with tunable parameters makes this procedure much more accessible to researchers who work primarily with well-characterized cell lines by facilitating use of high-confidence, phased VCF files produced by consortia such as GIAB. This frees researchers from the burden of having to generate suitable long-read data for read-based phasing and eliminate a large amount of redundant computation across groups.

Furthermore, many long-read assays currently in use and under development do not produce data suitable for read-based phasing. In particular, many assays use targeting to enrich for sequences meeting certain biological criteria. Since these data do not cover the entire genome, they likely violate many assumptions baked into current read-based phasing pipelines. Therefore, the quality of such phasing predictions is unclear, as is their suitability for any particular downstream analyses. Phasing reads within such datasets is likely more reliably performed using a high-confidence phased VCF from an institutional source.

These points have now been incorporated into the introduction.

- Along these lines: when considering haplotagging errors in practice, one would need to consider incorrectly phased variants as source of haplotagging error. Can you please address these points in the manuscript?

Additional discussion of variables likely to contribute to haplotagging error were added to the discussion (e.g., lines 814-819)

• The manuscript’s title states that LRPhase is efficient and configurable. However, efficiency is stated or quantified neither with respect to complexity nor with respect to practical runtime.

We performed benchmarking in our analysis, comparing run time and peak memory usage, for both LRphase and WhatsHap haplotag. However, the resulting metrics were difficult to compare owing to LRphase being implemented in Python while WhatsHap is implemented in C++. Thus, runtime comparisons were not meaningful and not reported.

That said, in our analysis of simulated data, we observed large differences in runtime, as expected, with WhatsHap haplotag clocking about 23 times faster than LRphase in User time (LRphase = 10216.72 seconds; WhatsHap = 440.21 seconds). This is consistent with expectations for speed gains when switching between python and C++, with the difference between the expected ~80-fold improvement and observed 23-fold difference owing to the greater computational complexity of the WhatsHap haplotag algorithm.

In contrast, peak memory usage for LRphase was substantially lower than WhatsHap haplotag, with LRphase using 138,336 kb at peak and WhatsHap haplotag using 801,684 kb. The difference is likely due to WhatsHap’s need to store an entire dynamic programming matrix for each read analyzed, whereas LRphase only needs to store a single log-likelihood value for each haplotype overlapping a given read.

We have added these stats to the manuscript, as requested (Lines 679-683).

• Further, the tool version 1.1.2 I tested had (except for mapping-related parameters) only the option to specify FDR or log likelihood threshold, which can both be considered a ranking of haplotagged reads,

Correct. In FDR threshold mode, the threshold log-likelihood-ratio is estimated based on the negative-binomial error model. If one knew this value ahead of time, supplying it as a log-likelihood threshold would produce equivalent results.

something that whatshap also allows (even though the authors mention they couldn’t find information on how to interpret the whatshap score).

After careful review of the WhatsHap haplotag documentation for the version used in the analysis (2.0), we are still unable to locate the option(s) to set an FDR threshold within whatshap haplotag. This does not appear to have changed in the latest version (2.1), based on documentation at https://whatshap.readthedocs.io/en/latest/guide.html. We invite the reviewer to share specific information on how this functionality is accessed. Meanwhile, we conjecture that the ability to do so may be provided by other utilities in the WhatsHap suite. If this is indeed the case, then it only serves to underscore our assertion that a standalone tool that includes meaningful ability to filter results is necessary to make haplotagging accessible to the broadest possible audience.

Please address and compare runtimes,

Done. See above.

state precisely adjustable tool parameters and

These are described in detail within the github README and we feel that reproducing them within the manuscript would introduce unnecessary clutter.

consider changing the manuscript title.
Concerns about the manuscript title are addressed below.

• There are two scoring modes mentioned, one of them simply maximizing the number of matching alleles. There are no results presented for this scoring and it is not discussed and put in relation to the newly described scoring. Is the statistical model considering error rates indeed better and in what settings?

After careful review of the code, we discover that Mode 2, as it is implemented, does not match the behavior described in the text. The actual behavior as implemented is to set a zero threshold on the log-likelihood ratios such that all reads with at least one heterozygous site are tagged. Since this is likely not mathematically equivalent to simply counting matches, we have removed mentions of the match-counting model from the text, figures, and supplementary materials. This mode is not available as a standard command-line option, and is now toggled by setting the log-likelihood ratio threshold to zero, as is more-appropriate to its actual behavior. Since this was a clear point of confusion, we have taken the time to clarify all scoring-model-related options in the HaplotagLR documentation.

• The scoring model is in my opinion the major contribution of this paper, but it is provided only in the supplement and not evaluated with respect to parameters or in comparison to “scoring mode 2”.

Since mode 2, as described in the text and figure 1, is no longer available, we have removed all mentions from text, figures, and supplementary materials.

W.r.t. the model, a sequencing read base error rate e_r[a] is defined. I think that including the base accuracy at the read’s variant position is very useful. However, it seems to not be used and only a global error rate epsilon is used that is neither estimated nor a tool parameter as far as I see.

We have clarified verbiage in the manuscript to indicate that, by default, epsilon is obtained as an average per read from the BAM record. However, the epsilon term can also be calculated directly from the Phred quality scores within each BAM read, or the user may supply a global value for epsilon, to be used in both scoring and error models. These options are now clarified in the documentation.

The effect of epsilon is not evaluated and epsilon cannot be adjusted in the LRphase version provided. Can you please clarify the notion behind the error rates, fix the corresponding notation and evaluate error rate effects?
The current version now offers the ability to fix epsilon at a global value.

• In the Supplement, some math notation is introduced, but not used (e.g. S), please fix this.
Fixed: we have removed two rows from the table in question that contained unused values.

• The entire Supplement should be moved to the main manuscript and carefully checked for notational correctness.
Relevant portions of the supplement, describing the scoring model, have been moved into the methods section.

• The model is nicely introduced in general terms for any ploidy in the Supplement, however, the main manuscript indicates that only a ploidy of 2 can be used (since it always refers to maternal and paternal haplotypes). Can the tool handle ploidy >2? If no polyploidy is implemented, this is a limitation that needs to be mentioned (whatshap can handle polyploidy!).
In principal, HaplotagLR can handle any given ploidy, not just 2. However, as the reviewer notes, this functionality is not fully implemented as of version 1.1.4. We acknowledge that WhatsHap does offer this functionality and plan to include it in a future release.

• Is the model indeed a new contribution? Similar ideas seem to have been applied in the context of haplotagging before, e.g. Pubmed ID 31873213. Relationship to previously published similar models in the context of haplotagging need to be worked out and stated.

After careful review of Pubmed ID 31873213, we discovered that the authors utilized WhatsHap haplotag for their haplotagging step. This is described in pg. 34, paragraph 3, of the associated supplementary information file, under the heading `Incorporating haplotype-specific sequencing reads (‘haplotagging’).` We found no evidence of any similar, novel model presented in this study. In fact, a rigorous literature review for terms “haplotag” and “haplotagging” failed to yield any additional reported tools or models for haplotagging. However, a related method with the same name is in common use among plant genetics researchers and this could be a source of confusion. If Reviewer 1 is aware of any additional published models for haplotagging as described here, we would love it if they would share them in any future responses.

• I find the naming of the tool, LRphase, misleading, since phasing is typically rather considered the phasing of variants, which this tool does not do, it only performs haplotagging. I think that it is important to reflect this in the tool name. What about LRtagging?

The reviewer is correct: LRphase does not perform phasing, so would be more accurately titled `HaplotagLR`. We have made the necessary changes in the manuscript and elsewhere.

• The description of related work is insufficient and needs additional attention. Only two tools are mentioned, whatshap haplotag and MarginPhase. With relation to their model, it is stated that they “appear to be based on the hidden-Markov model described in 15”. However, working out and stating the exact difference between the approaches and models and how this affects performance is important.

As far as we can tell, we have identified and reviewed all published materials relating to haplotagging as described in this manuscript. That said, we have also encountered a method sharing the same name that is used by the plant genomics community, but with very different scope and goals. Nevertheless, we have modified the text to reinforce the relationship between WhatsHap and MarginPhase, including their intended purposes and differences in the UI. To wit, we did not include MarginPhase in our comparison because it is implemented as a unified framework to perform read-based phasing and it lacks the ability to perform the haplotagging step (or any step, for that matter) in isolation.

**•** Whatshap seems according to Pubmed ID 36335496 very comprehensive and versatile. Thus, specific user-configurable parameters and output metadata reported by LRphase and not by Whatshap and how exactly they help in identifying haplotagging errors needs to be specified to support the proposed benefits of LRPhase.

This information is available on the github repository README.

When I tested LRphase as explained here ([https://github.com/Boyle-Lab/LRphase/tree/main/example_data](https://nam02.safelinks.protection.outlook.com/?url=https%3A%2F%2Fgithub.com%2FBoyle-Lab%2FLRphase%2Ftree%2Fmain%2Fexample_data&data=05%7C01%7Cadadiehl%40med.umich.edu%7C0aa22c2ef5174fe93a9508dbc356ea66%7C1f41d613d3a14ead918d2a25b10de330%7C0%7C0%7C638318550847931109%7CUnknown%7CTWFpbGZsb3d8eyJWIjoiMC4wLjAwMDAiLCJQIjoiV2luMzIiLCJBTiI6Ik1haWwiLCJXVCI6Mn0%3D%7C3000%7C%7C%7C&sdata=GZ2wsQu3QnmCpBGKbrQMT1KTGcRq7fNBuT1vuPFUn7o%3D&reserved=0) ), (i) I couldn’t find a documentation of the BAM file tags added by LRPhase and their interpretation (except HP for haplotype), (ii) I couldn’t find command-line parameters to adjust model parameters such as error rate, (iii)

In response to these points, we have added detailed documentation on tag values added by HaplotagLR and their meanings. This information is available on the github README.

no metadata output was produced that helps in investigating individual read haplotaggings.

All metadata are incorporated into BAM tags to obviate the need for an external metadata file.

According to my investigation of the tool documentation and the tool provided at bioconda, the current version 1.1.2 does not provide beneficial additional information or additional adjustable parameters except FDR.
Please see the github README for explanation of tunable parameters in the current version, 1.1.4. This information is also available by invoking HaplotagLR haplotag with the -h parameter.

• Please motivate why there is only one tool used for comparison, whatshap, and not at least also the other tool mentioned, MarginPhase.

See earlier comment on the MarginPhase UI, which does not provide the ability to haplotag as a standalone operation.

• No information is provided on runtime and there are no runtime comparisons with the other tool. This needs to be added.

As mentioned previously, a runtime comparison would not be meaningful since WhatsHap is implemented in C++ while HaplotagLR is implemented in Python. We would expect a >20-fold performance improvement if implemented in C++, and likely better performance than WhatsHap, since we are using a less computationally-complex scoring algorithm. All the same, we discuss it on lines 680-683 in the present revision.

• The overall approach is extensively explained in the caption of Figure 1. The approach should be rather more clearly explained within the main text and only for illustration shown in Figure 1.
Current version moves most verbiage from the figure 1 caption to the newly-added Haplotagging Algorithm and Scoring Model methods sections.

• Can you please comprehensively document the tool parameters and output specifications on the tool’s website?
We have attempted to clarify all user-available parameters for the program and believe we have not missed anything. Newly-added is a guide to the BAM tags added by HaplotagLR and how to interpret results.

• Can you please comment on the reliability of the haplotagging if the number of haplotagged reads is small? If simply lowest-scoring reads across an analyzed dataset are assigned to “unphased” to control FDR, then this is very sensitive to the number of reads being phased.
Reliability should not be affected by the number of reads, as log-likelihood ratios are calculated for each read independently of all others. Since the FDR threshold is used to calculate a number of reads to truncate as a fraction of the total number of reads, then the number of reads to truncate will evaluate to <1 below a certain threshold as the number of input reads decreases. In these cases, the error model has no impact on empirical FDR. This is a flaw we now acknowledge in the discussion (lines 812-814)

• How is the average sequencing error rate estimated? How is the output interpreted? Some tool parameters are not documented in the help, e.g. the mandatory (?!) parameter -s. Can multiple samples be processed at once? Please improve the documentation with respect to these points.

The default value for the average sequencing error rate, epsilon, is calculated as the mean observed sequencing error rate for all reads processed. This is now indicated in the text (lines 432-433)

This behavior mimics what happens during scoring: the sequencing error rate is determined directly from the BAM records whenever possible.

HaplotagLR is under active development, thus arguments and requirements are evolving. The current version now removes the requirement for the -s argument, instead retrieving the sample name from the VCF input file. The present version can only handle a single VCF sample, which is used to process all reads in the BAM input. We are actively developing a version that will handle multiple samples in both VCF and BAM.

This planned version will also provide access to the global sequencing error rate, epsilon.

• Can you please provide the scripts used to rescue the GIAB VCF file and to simulate the read data?
Done. See above.

• The notation of manuscript (especially Fig. 1) and model description (now Supplement) should be harmonized
Done

• Many math symbols don’t show in the PDF and I cannot check them. This should be fixed. You may consider switching to Latex, especially since the formulas in the Supplement render poorly and are partly inconsistent in font.

Hopefully this is resolved in the current revision. We have attempted to remove any non-standard fonts and characters from all figures and equations. However, since we cannot see the converted version, we cannot be certain that all are fixed. Converting the manuscript to latex at this late stage would be unreasonable.

Reviewer #2: In this work, the auhtors present LRphase, user-friendly tool that haplotags long sequencing reads based on a multinomial model and existing phased variant lists.
The overall result of the manuscript is very promising. The manuscript is overall readable.
The topic is interesting and it is well explained as well as the work. The paper presents some issues to be improved.

First, the introduction should focus on the goal of the work that it is not clear. Furthermore, from the state of the art it is not possible to understand if there are other similar works.
We believe we have identified all related works. However, we would be open to incorporating any we have missed if the reviewer would kindly share references.

The authors should add a related work section.
This is captured in the discussion section.

Have they tried other state-of-the-arts methods ? If so, how were the results? If not, what was the reason?
This was addressed in responses to reviewer #1. Namely, of the two available methods, only WhatsHap offered a suitable workflow for comparison, whereas MarginPhase did not offer the ability to haplotag as a standalone method.

The authors should add a comparison section.
Comparisons to WhatsHap are already given in the results section. We fail to understand how moving these results to a separate section would improve clarity, since they already form the majority of the Results section. Nevertheless, we have added section headers within the main results section that we hope will improve the manuscript’s flow and readability.

Finally, the discussion of the results should be improved.
This suggestion is very imprecise, so we do not know exactly how to respond. We hope that edits made in response to reviewer #1 will yield the needed improvements.

About the conclusion, the author should describe what are the future applications of research and the practical advantages.

This is addressed in the final two paragraphs of the current Discussion section.
