# Supplementary material for: HaplotagLR: An efficient and configurable utility for haplotagging long reads
Source: PLoS One. 2024 Mar 13;19(3):e0298688. doi: 10.1371/journal.pone.0298688 (PMC10936807; doi:10.1371/journal.pone.0298688)
Supplement: Attachment — Submitted filename: LRphase_PLOS-One_rebuttal-letter.rev2.docx [file pone.0298688.s003.docx]

Black text: Reviewers' comments
Red text: Authors’ responses

Reviewer #1: The authors have properly addressed most of my concerns and I believe the manuscript and tool documentation improved significantly.
Remaining points are:

• The presentation of the model (section scoring model) is still poor and the formulas in the manuscript and supplement are inconsistent, e.g. in the supplement a posterior probability beta is introduced. Which formulas are correct? Please don’t put the mathematical introduction of the problem in the supplement. I still think that all supplementary content (mathematical introduction of model background phase sets, VCF etc.) should be moved to the main manuscript.

We have moved all supplementary information into the main text in the current revision and believe we have addressed all notational mismatches in the process.

When doing so, please remove the i (relating to an individual read) which is now used in the manuscript formulas, it makes the formulas unnecessarily complicated.
See above.

• Typo at “Therefore, a haplotag based on variant l may be reduced to a random guess”
Fixed.

• The epsilons (?) in section” Haplotagging Error Model” are still rendering incorrectly
The reviewer is correct that the notation in question should display as lower-case greek letter epsilon; we are unsure as to why these symbols are not rendering properly for the reviewer.

We believe we are properly interpreting and following the guidelines given at (https://journals.plos.org/plosone/s/submission-guidelines) for in-line mathematical notation:

*“Avoid using MathType, Equation Editor, or the Insert→Equation function to insert single variables (e.g., “a² + b² = c²”), Greek or other symbols (e.g., β, Δ, or ′ [prime]), or mathematical operators (e.g., x, ≥, or ±) in running text. Wherever possible, insert single symbols as normal text with the correct Unicode (hex) values.”*

We have verified that these symbols show up properly on screen, when printed, and when saved as a PDF, using the .docx document and Microsoft Word 16.79.2.

• The script is not available from the link provided in the manuscript: “The rescued and filtered VCF and scripts to produce it from the original are archived at [https://zenodo.org/record/7819303#.ZDW6sexudzU](https://nam02.safelinks.protection.outlook.com/?url=https%3A%2F%2Fzenodo.org%2Frecord%2F7819303%23.ZDW6sexudzU&data=05%7C01%7Cadadiehl%40med.umich.edu%7Cc769d3f5453f43cf456608dbf36545f5%7C1f41d613d3a14ead918d2a25b10de330%7C0%7C0%7C638371388520696855%7CUnknown%7CTWFpbGZsb3d8eyJWIjoiMC4wLjAwMDAiLCJQIjoiV2luMzIiLCJBTiI6Ik1haWwiLCJXVCI6Mn0%3D%7C3000%7C%7C%7C&sdata=unSEOkqOBEvS61W4VL17T31kqH8DzXJtParOk05UjU8%3D&reserved=0)”

Regrettably, the scripts were mistakenly added to the incorrect repository in zenodo in responding to previous comments. We have now fixed this and the above repository now includes all data and scripts as-described.

• Can you please improve the github documentation of “-e EPSILON, --epsilon EPSILON” and “-c, --epsilon_from_quality_scores” and be more specific in the manuscript: There are three settings discussed: (i) global error rate provided by user, (ii) global error rate as mean phred-score deduced from error rate across the entire read, (iii) every base has an individual error rate, computed from the base’s PHRED score. I think (iii) is by far the most reasonable. However, I cannot follow, which setting is which and whether all three or only two are implemented in the tool.

All three modes are implemented as described. The default mode, which is assumed when neither –e or -c are supplied as arguments, calculates epsilon as a mean per-read. Both -e (--global_epsilon – formerly –epsilon) and -c (--epsilon_from_quality_scores) arguments may be used to override this default mode, with –global-epsilon taking highest priority if both are supplied. We have edited the documentation to clarify how these options relate to the model as described in the manuscript.

• I was not able to install the tool via conda using the command from the github documentation due to unresolvable incompatibilities, please fix that. Installation with pip worked.

This seems to have been caused by the (unnecessary) dependency on python 3.7 in the conda install command as presented on github. We have removed this from the current documentation.

• The peak memory usage is for both compared tools negligibly small, which should be stated.

Done.
